# Genetic Alterations Featuring Biological Models to Tailor Clinical Management of Pancreatic Cancer Patients

**DOI:** 10.3390/cancers12051233

**Published:** 2020-05-14

**Authors:** Shannon R. Nelson, Naomi Walsh

**Affiliations:** National Institute for Cellular Biotechnology, School of Biotechnology, Dublin City University, Dublin 9, Ireland; shannon.nelson5@mail.dcu.ie

**Keywords:** pancreatic cancer, GWAS, genomics, organoids, cancer models

## Abstract

Pancreatic ductal adenocarcinoma (PDAC) is the fourth leading cause of cancer-related death worldwide. This high mortality rate is due to the disease’s lack of symptoms, resulting in a late diagnosis. Biomarkers and treatment options for pancreatic cancer are also limited. In order to overcome this, new research models and novel approaches to discovering PDAC biomarkers are required. In this review, we outline the hereditary and somatic causes of PDAC and provide an overview of the recent genome wide association studies (GWAS) and pathway analysis studies. We also provide a summary of some of the systems used to study PDAC, including established and primary cell lines, patient-derived xenografts (PDX), and newer models such as organoids and organ-on-chip. These ex vitro laboratory systems allow for critical research into the development and progression of PDAC.

## 1. Introduction

With a five-year survival rate of 9%, pancreatic cancer has one of the worst outcomes of all cancers. Due to its rapid progression and fatal outcome, long-term survivors are limited to those with resected early-stage tumours [1,2]. As 80% of pancreatic cancer patients are diagnosed after the disease has metastasized, most people diagnosed are ineligible for resection, the only curative treatment. It is the fourth leading cause of cancer-related death in the Western world, and by 2030 it is estimated that pancreatic cancer will surpass breast and colorectal cancer to become the second most fatal cancer in the United States [3]. The most common form of pancreatic cancer is pancreatic ductal adenocarcinoma (PDAC), which occurs in the exocrine pancreas, with the remaining 5% of cases in the endocrine pancreas [4]. Epidemiological factors, including smoking, obesity, type II diabetes mellitus and acute pancreatitis account for approximately 25% of cases of PDAC [5,6,7,8].

The mean survival time of patients who receive the surgery and adjuvant treatment is 11 to 23 months. Of the patients who are operated on, 60% relapse within 12 months; this is most likely due to micro-metastases which were not detected during the diagnostic computed tomography (CT) scan [9]. Approximately 25–30% of patients treated with chemotherapeutic drugs respond, however most eventually become resistant. Resistance mechanisms include deficiencies in drug uptake, alteration of drug targets, activation of DNA repair pathways, and resistance to apoptosis [10]. Gemcitabine is the mainstay of modern chemotherapy for pancreatic cancer [11]. FDA approval has also been granted for gemcitabine in combination with erlotinib and paclitaxel in 2005 and 2013 respectively [12]. The drug combination FOLFIRINOX (irinotecan, oxaliplatin, 5-fluorouracil, and leucovorin) was approved by the FDA in 2011.

## 2. Genomic Variants of PDAC

In addition to epidemiological factors which account for 25% of cases, research into the genetic landscape of the disease, including familial cancer syndromes, inherited predisposition loci and somatic mutations is vital to identifying those at risk of developing the disease.

### 2.1. Familial Cancer Syndromes

Familial cancer syndromes including Peutz-Jegher Syndrome (PJS), pancreatitis, familial atypical multiple mole and melanoma syndrome (FAMMM), Lynch syndrome, Hereditary Breast and Ovarian Cancer (HBOC) syndrome and Familial adenomatous polyposis (FAP), account for approximately 5–10% of pancreatic cancers. Table 1 contains an outline of the diseases and syndromes associated with an increased risk of developing PDAC.

Peutz-Jegher Syndrome (PJS) is a rare autosomal dominant disease, characterised by gastrointestinal polyposis, mucocutaneous pigmentation, and cancer predisposition [13]. PJS increases the risk of several malignancies, including breast, pancreatic and gynaecological cancers [14]. Individuals with PJS have a 132-fold increased risk of developing PDAC [15]. It is caused by a mutation in *STK11*, also known as liver kinase B1 (*LKB1*). *STK11*/*LKB1* is a serine/threonine protein kinase which drives many cell functions, including cell growth, regulation of metabolism and cell polarity, mainly through AMP-activated protein kinase/ mammalian target of rapamycin (AMPK/mTOR) signalling [16]. The most common *STK11*/*LKB1* mutations are deletions or inactivating mutations. In a genetically engineered mouse model (GEMM) study by Helez et al. [17] *STK11*/*LKB1* deletion resulted in defective acinar cell polarity, with abnormal cytoskeleton, loss of tight junctions, and progressive acinar degeneration. Deletion of *STK11*/*LKB1* in the pancreas also resulted in the development of serous cystadenomas. Morton et al. [18] showed that the *STK11*/*LKB1* deletion resulted in accelerated *KRAS*^G12D^ tumorigenesis, through decreased *TP53* and *p21* dependent growth arrest. These studies, along with others provide strong evidence for a tumour suppressor function for this gene [19].

Pancreatitis is the second most common hereditary cause of PDAC. Pancreatitis is an inflammatory disorder of the pancreas, caused by the premature activation or lack of inhibition of digestive enzymes. There are several forms of hereditary pancreatitis, including a gain of function mutation in serine-1 protease gene (*PRSS1*), which makes trypsinogen [20]. This gain of function mutation results in increased trypsinogen auto-activation, which triggers pancreatic self-digestion. Other genes associated with hereditary pancreatitis include *SPINK1*, a pancreatic secretory trypsin inhibitor and *CFTR* (cystic fibrosis transmembrane regulator) [21]. The chronic inflammation of the pancreas which characterises pancreatitis result in the presence of reactive oxygen species (ROS) in the pancreas [22]. These ROS, including nitric oxide and free radicals inhibit apoptosis, and can result in direct DNA damage, resulting in oncogenic mutations in genes such as *KRAS*, *CDKN2A* and *TP53* [23,24]. Cytokines which are released in response to pancreatitis activate pancreatic stellate cell and result in the development of fibrosis, facilitating the development of PDAC [25,26,27]. People with chronic or hereditary pancreatitis have a 69-fold increased risk of pancreatic cancer [28].

An autosomal dominant disorder, familial atypical multiple mole and melanoma syndrome (FAMMM) is characterised by melanoma in more than one first- or second-degree relative, high total body mole count (often >50), and moles with certain histopathological features. The melanomas can arise from the atypical moles or de novo, superficially spreading melanoma and/or nodular melanoma [29]. Three original descriptions in different kindreds implicated germline mutations or microdeletions in cyclin-dependent kinase inhibitor 2A (*CDNK2A*), in particular the p16^INK4a^ isoform, as causative for FAMMM [30]. FAMMM results in a 13 to 22-fold increased risk of PDAC. *CDKN2A* is also mutated in 90–95% of sporadic PDACs [31,32]. It inhibits cyclin dependent kinases 4/6 (*CDK4*/*CDK6*) and thereby activates the retinoblastoma (RB) family of proteins, which blocks the transition from G1 to S-phase [33]. It is mainly associated with the autosomal dominant familial melanoma, but patients also have an increased risk of PDAC [34]. By identifying individuals with an increased risk of developing PDAC from a family history, or families with a gene defect which results in PDAC, Vasen et al. [35] detected PDAC in 7.3% of the *CDKN2A* mutation carriers, by providing an annual Magnetic Resonance Imaging (MRI) scan, resulting in a resection rate 75% and an overall 5-year survival rate of 24%.

Lynch syndrome is also associated with an increased risk of colorectal cancer and PDAC [36]. It is caused by mutations in the mismatch repair genes (MMR), mainly MutL homolog 1 (*MLH1*), MutS homolog 2/6 (*MSH2*/*MSH6*) and PMS1 Homolog 2 (*PMS2*). The MMR maintains the integrity of the genome by repairing DNA replication errors [37]. Bi-allelic loss of MMR genes results in genomic instability, and an increase of unrepaired replication errors, particularly affecting repeats, such as microsatellites, termed microsatellite instability-high (MSI-H). MSI-high results in genome hypermutability, with a 100- to 1000- fold increase in mutations [38,39]. Individuals with Lynch Syndrome have 8.6-fold increased risk of PDAC [40].

Hereditary Breast and Ovarian Cancer syndrome is caused by mutations in the tumour suppressor genes *BRCA1* and *BRCA2*. People with this syndrome have a 3.5–10-fold increased risk of developing PDAC [41]. Mutated variants of the Partner and Localiser of BRCA2 (*PALB2*) gene are also associated with a familial risk of PDAC [42]. *PALB2* has a critical role in homologous recombination repair (HRR) and recruits *BRCA2* and *RAD51* to DNA breaks. Jones et al. [43] found that in 96 patients with PDAC, three had truncating mutations in the *PALB2* gene, producing a stop codon, which was not present in 1084 healthy controls. Slater et al. [44] observed a similar prevalence of *PALB2* mutations (3.7%) in a panel of 81 European patients with familial pancreatic cancer. A study in a 61-year-old patient with advanced localised PDAC, with a bi-allelic inactivation of *PALB2*, found treatment with Mitomycin C resulted in disease regression, and at the 3-year follow up, the patient remained asymptomatic. Studies also showed that patients with wild type *PALB2* are Mitomycin C resistant [45].

Familial adenomatous polyposis (FAP), is a familial cancer syndrome which results in an increased risk of colorectal cancer [46,47]. It is characterised by colorectal polyps, due to a mutation in the adenomatous polyposis coli (*APC*) gene. *APC* acts to negatively regulate the Wnt signalling pathway [48]. The Wnt proteins stabilise cytosolic β-catenin, which associates with the transcriptional regulators T cell factor/lymphoid enhancer factor-1 family (TCF), thereby allowing the expression of Wnt-regulated genes [49]. Murine studies of colorectal cancer have found that mutations in the *APC* gene result in hyperproliferation of cells [50]. Individuals with FAP have 4.5 to 6-fold increased risk of PDAC [47].

### 2.2. Inherited Predisposition Loci

Recently, the landscape of pancreatic cancer has been redefined through gene expression and genetic diversity signatures identified using next generation sequencing (NGS) and genome wide association studies (GWAS). GWAS examines hundreds of thousands of variants, in thousands of individuals, to identify genotype-phenotype associations, and helps to identify risk factors for multifactorial diseases [51]. Through this, GWAS can enable the identification of people at risk of developing a disease, and also can be used for the examination of the biological underpinnings of a disease. GWAS enables the use of potential preventative measures for those who are identified as at risk, and also for the development of treatments for the disease. GWAS use single nucleotide polymorphisms (SNPs) which are single base pair changes in the genome. SNPs can occur in the gene, in both introns and exons which result in amino acid changes, different mRNA splicing and reduce the mRNA transcript stability [52]. SNPs can also occur in the transcriptional regulatory elements such as transcription factor binding sites, enhancers and promoters, resulting in altered mRNA expression [53].

**Figure 1:**
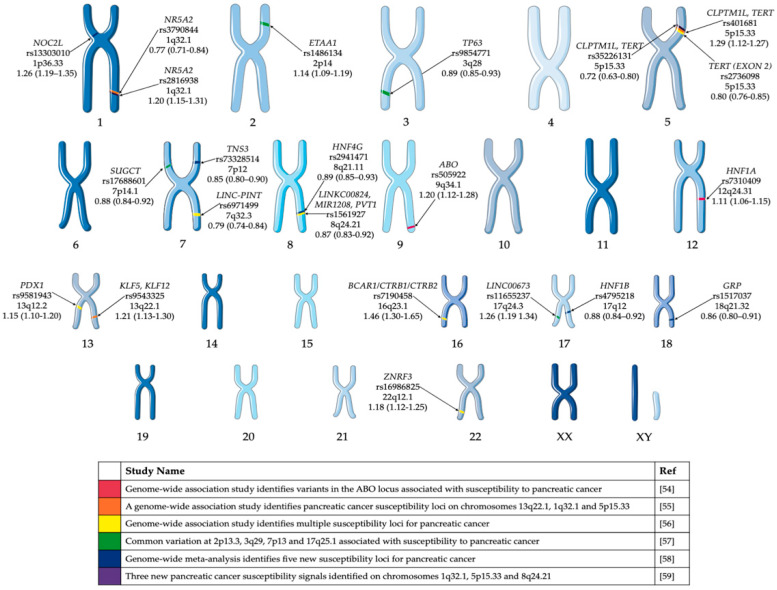
GWAS significant single nucleotide polymorphisms (SNPs) identified in pancreatic cancer cases of European ancestry. Highlighted GWAS SNP, closest gene, chromosome and odds ratio (95% confidence interval) [54,55,56,57,58,59]. This figure was created using Servier Medical Art templates, which have been modified. These images are licensed under a Creative Commons Attribution 3.0 Unported License; https://smart.servier.com.

Several PDAC GWAS studies have been performed over the past decade [54,55,56,57,58,59] and have identified common variants associated with risk of pancreatic cancer in European populations (Figure 1). Obazee et al. [60] used the PANDoRA dataset to validate a truncating *BRCA2*^k336X^ (rs11571833) and pathogenic *CHEK2*^I157T^ (rs17879961) variants. Both genes are critical in DNA repair and the maintenance of genomic stability. While the results of the GWAS have informed the genetic component of predisposition loci, it does not give a clear indication of the cause of PDAC. The use of complementary GWAS pathway analysis—a method of analysing this genomic data through sets defined by functional pathways—offers the potential of greater power for discovery and natural connections to biological mechanisms. Pathway analysis allows for the identification of causative SNPs whose individual effects may not be significant enough to be detected in GWAS [61]. Pathway analysis of PDAC GWAS SNP data has been previously performed [62,63]. Walsh et al. [63] performed a pathway-analysis based on meta-analysis of PDAC GWAS. Pathways associated with the development of the pancreas, including pancreas development and the regulation of beta cell development were among the pathways identified in these studies (Table 2).

A recent study by Campa et al. [64] looked at the genetics of early onset pancreatic cancer (EOPC)—disease which occurs in those sixty-years or younger, and represents 20% of cases of PDAC. Four SNPs (rs7155613, rs2328991, rs4891017 and rs12610094) were found to be associated with EOPC (*p* < 1 × 10^−4^). Of the SNPs identified, rs2328991 at 13q22.3 was found to be significant in the replication dataset. The SNP is 57 kb from the 3′ UTR of the potassium channel tetramerization containing 12 gene (*KCTD12*) which has previously been implicated in gastrointestinal stromal tumours.

### 2.3. Somatic Mutations

**Figure 2:**
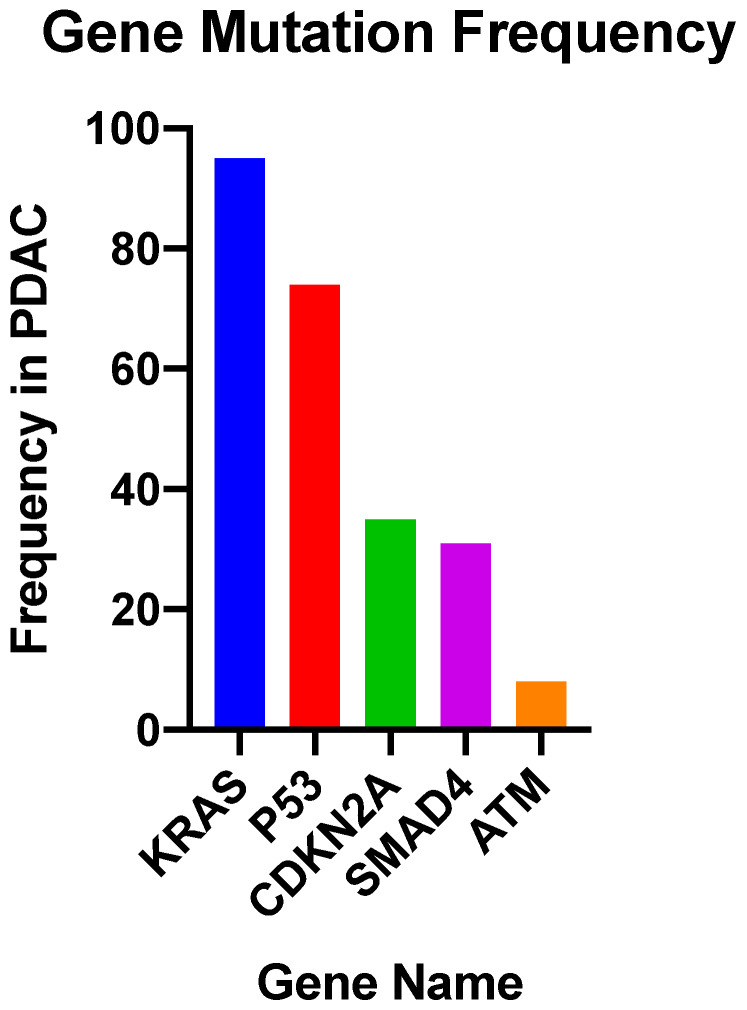
Most common somatic common gene mutations in PDAC [67,68].

Studies into the PDAC genome have shown that there are approximately 60 alterations per tumour; most of which are point mutations [65]. Activating mutations of *KRAS* are nearly universal, and inactivation of *TP53, SMAD4* and *CDKN2A* occur at rates of >50% (Figure 2) [66].

*KRAS* is a molecular switch, when bound to GTP regulates cell proliferation, differentiation, apoptosis and cell signalling. The *KRAS* mutation is near universal in PDAC, with 94% of tumours possessing the mutation [68]. Activating point mutations in codon 12, 13, or 16, (most commonly G12D), result in reduced GTP hydrolysis. Cases with *KRAS* mutations at codon 61 give a favourable prognosis, as there is less ERK activation [69]. *KRAS* mutations in pancreatic cancer are believed to be the early events in neoplastic transformation [70]. Oncogenic *KRAS* is not sufficient to initiate the carcinogenesis process, this relies on the downstream activation of *Raf-1*, *Rac*, *Rho* or *PI3K* [71]. RAS proteins are modified by farnesyl transferase, an enzyme which adds 15-carbon farnesyl lipid to the carboxyl-terminal cysteine of RAS. This modification is shown to be essential for RAS membrane association and transformation [72]. A Phase III clinical trial of R115777, a selective inhibitor of farnesyl transferase combined with gemcitabine failed to show increased life expectancy in comparison to gemcitabine plus placebo [73]. Patients with *KRAS* mutations were associated with a median survival time of 17 months compared to 30 months for those without mutations [70]. Approximately 3% of PDAC cases are due to microsatellite instability or altered chromosome ploidy [74]. This is usually due to mutations in the MMR genes *MSH2* and *MSH6*. Typically, *KRAS* is wild-type in cancers with these mutations.

*CDKN2A*, previously discussed as the causative gene of FAMMM, is inactivated in 95% of PDAC cases, by homozygous deletion, mutation of alleles or promoter hypermethylation resulting in gene silencing [75].

*TP53* is the most frequently mutated gene in cancer [76]. It acts as a tumour suppressor, and has roles in apoptosis, genomic stability, inhibition of angiogenesis and arrest of cell growth [77]. It is mutated in 75% of PDAC tumours, mainly by point mutations [78]. TP53 controls cell cycle at the G1/S interface and plays a vital role in inducing programmed death in response to DNA damage [1]. Weissmueller et al. [79] found mutant *TP53* induced platelet-derived growth factor receptor b *(PDGFRb).* Knockdown of *PDGFRb* in PDAC cell lines resulted in reduced invasion of the cells. Mutant *TP53* inhibits *p73*, which represses *PDGFRb.* The study found that increased expression of *PDGFRb* in PDAC patient samples correlates with a worse outcome for patients.

*SMAD4* is a transcription factor in TGFβ signalling pathway and is inactivated in 50% of advanced pancreatic cancers. It acts with TGFβ1 as a tumour suppressor to regulate pancreatic cell cycle arrest, and apoptosis, mediated by targets such as p21, which causes G1 cell cycle arrest [80]. Patients with biallelic deletion of *SMAD4* more frequently had metastasis than those with wild type *SMAD4* [81].

Whole genome sequencing of PDAC identified other genes which are frequently mutated in PDAC, such as ataxia telangiectasia mutated (*ATM*), a serine/threonine kinase with a role in DNA double strand break repair; and pathways including the TGFβ, the β-catenin and Notch pathways [58]. Recent data based on large-scale sequencing studies reported up to 18% of *ATM* mutations in PDAC cohorts [65,67,68,82,83].

## 3. Models of PDAC Research

### 3.1. Established PDAC Cell Line Cultures

Two-dimensional, cell-based assays are an important tool, and have been the mainstay of cancer research for over 50 years. The first cell line (HeLa) was developed in 1950 from cervical carcinoma [84]. Cell lines are able to grow indefinitely, making them an easy to use, low cost, repeatable model, and thus important for both drug discovery, and proof-of-concept studies. While the usefulness of cell lines in cancer research is certain, their use as a clinical model is debatable [85]. Cell lines often undergo genetic modifications, including copy number variation and point mutations during passaging [86]. Cell lines tend to be homogeneous which does not represent the heterogeneous nature of PDAC tumours. Cell lines are often developed from late stage, aggressive tumours, so they cannot be used to model tumour progression [87].

PDAC cell lines recapitulate the genomic changes which lead to the development of the disease. The four most common mutations (*KRAS*, *TP53*, *CDKN2A SMAD4*) occurring in PDAC tumours are found in cell lines at similar percentages and PDAC cell lines also demonstrate the different phenotypes and genotypes which are found in PDAC subclasses. A commonly used PDAC cell line is BxPC3, developed from pancreatic adenocarcinoma of a 61-year old female. BxPC3 has *TP53* mutations, a homozygous deletion in *SMAD4*, but is *CDKN2A* wild type and is the only *KRAS* wild type PDAC cell line. Other common PDAC cell lines include, PANC-1, developed from PDAC of a 56-year old male and MIAPaCa-2, developed from a PDAC of a 65-year old male, harbour mutations in *KRAS* and *TP53*, with homozygous deletion in *CDKN2A* and wild type *SMAD4*. Capan-1 was developed from a liver metastasis of a 40-year-old male with PDAC, and harbours mutations in *KRAS*, *TP53*, *CDKN2A* and *SMAD4,* and is the only PDAC cell line with a *BRCA2* mutation. A detailed review from Deer et al. [88] provides an overview of the available information on the most commonly used PDAC cell lines.

Due to genomic drift, differences in cell culture procedures and media, in different labs may result in genotypic and phenotypic differences in the same cell line. Recently, Ben-David et al. [89] performed a full genomic characterisation of 27 different strains of the ER-negative breast cancer cell line MCF7. Changes were observed including differential activation of gene expression programs, morphology and proliferation. Drug sensitivity was shown to vary in the cell lines, with 75% of the drugs that were tested which strongly inhibited some of the MCF7 cell lines, were completely inactive in others.

Another issue with the use of established cell lines include cross contamination. Boonsta et al. [90] have identified two oesophageal adenocarcinoma cell lines which were contaminated, and have been used in 11 patents, and more than 100 published studies, leading to clinical trials. Horbach and Halffman [91] identified 32,755 articles reporting on research with misidentified cells, which in turn have been cited by over half a million papers. To overcome these issues, a number of journals require cell lines to verify before publishing a research paper. The method used to validate the cell lines are short tandem repeat (STR) profiling. These techniques were initially developed for forensic applications [92]. STR profiling compares microsatellite (2 to 7 base pairs) repeats at specific loci which are unique to each individual [93]. It is carried out by using commercially available PCR primers which are compared to size markers, allowing for a comparison of the lengths of the PCR products at each locus to the STR profile made from the original donor material [94].

Two-dimensional (2D) established cell line models have been standard method for cancer drug testing for many years, however, of late, the limitations of using established cell lines in 2D are being increasingly recognised. In actuality, 2D cell culture platforms often fail to recapitulate the physiology of tumours in vivo due to different cellular architecture, adherence structures and biochemical gradients.

### 3.2. Primary Cell Lines

Primary cell lines are an emerging tool for cancer research. These cell lines are derived from a patient tumour or biopsy, dissociated, and grown in vitro [95]. Primary cell lines are heterogeneous, and are at an early passage number, so are more representative of the original tumour [96]. Primary cell lines may allow for the development of personalised cancer therapy through the development of primary cell lines from patient tumour, and the function testing of chemotherapeutic drugs on the living cancer cells [97]. While primary cultures are more representative of the original patient tumour, there are several issues with PDAC primary cell lines—they are often difficult to establish, only grow for a limited number of passages, and often tumour cells are overgrown by stromal cells such as fibroblasts [96,97].

### 3.3. Organ-on-Chip

“Organ-on-chip” is a microfluidic chip containing multiple cell types which are joined by microchannels and can simulate the activities of entire organ-systems. These cancer models can be used to represent the tumour microenvironment, and can show cancer initiation and progression, observations of the interactions and signalling pathways of different cell types [98]. This model can be used to identify potential metastasis sites of the tumour, observed the impact of immune cells in cancer, and to determine the effects of cancer treatment on other organs [99,100].

Beer et al. [101] used HepaChip organ specific 3D cell culture chambers to culture PDAC cell lines PANC-1, BcPC3 and MIAPaCa-2 with the extracellular matrix protein collagen. The cells maintained the viability, morphological appearance, and growth characteristics of 3D spheroids when grown on a chip.

Using an organ-on-chip model, Nguyen et al. [102] studied tumour-endothelium in PDAC, which is a poorly vascularised cancer. PDAC 3D organotypic models were placed in a chamber next to endothelialised, perfused lumen. The study found that through the TGF-β receptor signalling pathway, activin-ALK7 allowed for endothelial ablation, where the PDAC cells invaded and removed the vascular endothelium, leaving tumour filled structures. These results were then validated in vivo.

### 3.4. Patient Derived Xenografts (PDX)

Patient derived xenografts (PDX) are another commonly used model of PDAC. Patient tumour is implanted subcutaneously or orthotopically in severe combined immune deficiency (SCID) mice until the tumour has grown to a sufficient size to be sub-cultured in new mice. These models allow for tumours to have the original cell-to-cell interactions [103]. The original tumour microenvironment can also be recapitulated using orthotopic implants. A study by Garrido-Laguna et al. [104] showed that orthotopic implantation closely mirrors the results from the clinic. Orthotopically implanted tumours treated with gemcitabine had a similar response to that in patients, which was not observed in the subcutaneous implanted tumours.

PDX studies have been used for the identification of biomarkers of PDAC. Jimeno et al. [105] used 11 PDX tumour samples, with known gemcitabine sensitivity to identify biomarkers for gemcitabine response in patients. This group exposed fine-needle biopsy of the PDX tumour to gemcitabine or vehicle control for 6 h and compared gene expression of the treated and untreated samples using qRT-PCR 45-gene array. This assay identified that Polo-Like Kinase 1 *(Plk1)*, a serine/threonine-protein kinase had differential expression of >50% in the sensitive samples compared to resistant tumours. To further validate this biomarker, the group performed siRNA knockdown and inhibition of the Plk1 pathway using a pathway modulator which resulted in a synergistic effect with gemcitabine in gemcitabine-resistant in vitro models. The study illustrates the ability to use PDX models to identify and validate a biomarker of PDAC.

There are many advantages to using PDX tumours for the study of pancreatic cancer. Tumours can be established in mice using a small amount of tumour; tumours retain the heterogeneity, as well as the genetics, and histological characteristics of the original tumour during passaging. PDX tumours also provide an unlimited source of tumour, which can be used for in vivo and ex vivo drug testing. Nevertheless, there are several disadvantages to the use of PDX models—they are expensive, time consuming, require the use of animals, and their use is subject to strict regulations [106]. PDX models take up to four months to develop tumours. Subcutaneously implanted tumours are not grown in the same microenvironment as PDAC tumours, and rarely form metastases [107]. As the tumour is grown, and sub-cultured in mice, the human stromal cells, such as fibroblasts and blood vessels are replaced by murine cells [108]. Finally, as SCID mice do not have an immune system, the PDX tumours cannot recapitulate the complex interactions between the PDAC tumour and the immune system, which is critical in resistance mechanisms of PDAC, and it also prevents the use of PDX models in testing of immune modulating drugs, which are increasingly being used in cancer treatment.

### 3.5. Genetically Engineered Mouse Models (GEMM)

Genetically engineered mouse models of PDAC can be used for both basic and translational cancer research. GEMM develop *de novo* tumours in an immune proficient environment, mimic the histopathological and molecular features of human tumours [109]. They also spontaneously develop metastatic disease [109]. With the use of CRISPR genome editing technology allowing for site directed double strand breaks resulting in gene knockouts and the introduction of defined mutations, the development of GEMM has become easier [110]. The KRAS^LSL.G12D/+^; Pdx-1-Cre (KC) inducible knock-in GEMM, presents with slow disease progression, results in the development of Pancreatic Intraepithelial Neoplasia (PanIN). At a low frequency, these PanINs can also develop into locally invasive, and metastatic adenocarcinoma, allowing for the use of the KC model to study PanIN development and strategies to delay PDAC [111].

In order to overcome the challenges in studying immune-related drugs, GEMM contribute to immune research in PDAC. These models most include the most commonly mutated genes in PDAC, such as *KRAS*, *TP53*, *SMAD4* and *CDKN2A*. The *LSL-Kras^G12D/+^*; *LSL-Trp53^R172H/+^*; *Pdx-1-Cre* (KPC) model is one of the most commonly used models for studying immunotherapy in PDAC. This model has the same features of the immune microenvironment as human PDAC, including the exclusion of effector T-cells [112]. The KPC model utilises a Cre-Lox technology, with the *KRAS*^G12D^ and *TP53*^R172H^ mutations, with the Cre-recombinase activated by pancreas specific transcription factor PDX1. In this model, the new-born mouse has a normal pancreas, with PanIN development beginning at 8–10 weeks, with epithelial to mesenchymal markers such as decreased expression of E-cadherin, and increased expression of *Zeb1* and *Fsp1*. At 16 weeks, the mice have developed locally invasive PDAC and the mice display cachexia, jaundice, weight loss malignant ascites and metastases. The KPC PDAC mouse model has been used in many pre-clinical studies, including Olive et al. [113] who studied the co-administration of gemcitabine and IPI-926, a drug which inhibits the Hedgehog signalling pathway to deplete tumour associated stroma, which resulted in an increased intertumoral concentration of gemcitabine. Frese et al. [114] used the model to study the effect of paclitaxel in combination with gemcitabine, and found increased intertumoral levels of gemcitabine due to the decreased levels of cytidine deaminase, which metabolises gemcitabine.

## 4. Organoids

The use of three-dimensional (3D) in vitro PDAC models can overcome many of the limitations of traditional cancer research models. As they are not attached to plastic, 3D models have more appropriate physiological morphology and signalling pathways compared to cells grown in 2D [115]. Similar to in vivo conditions, 3D cultures are exposed to complex environments, with varied exposure to oxygen, nutrients, stress and waste. The use of 3D cultures also allows for the study of cell-to-cell interaction; drug penetration, response and resistance [116,117]. Another advantage of using 3D models, is the cultures contain cells in multiple growth phases, with cells which are proliferating, quiescent, hypoxic and necrotic cells, whereas cells grown in 2D tend to be in the same growth phase [118]. Thus, 3D models have the same gene and protein expression profiles as the original tumour whereas differential expression is present in 2D cell models [119,120,121]. Cells which are grown in 3D can also be cultured and tested for longer, as 2D cells require regular trypsinisation as cells reach confluency faster [122]. Previous studies have shown that cells grown in 2D and cells grown in 3D have different sensitivity levels to chemotherapeutic drugs, with 3D models showing increased levels of drug resistance, which is more representative of the in vivo drug response [123,124].

The first publication describing intestinal organoids was published in 2009 [125], and since then, the methods have been used to create organoids in a large range of tissues and to study a wide range of diseases. Organoids are 3D spheroid cultures which represent the in vivo architecture of the organ or tumour. Organoids are developed from stem cells, which self-organise to resemble tissues from within the organ. They can be derived from multiple types of stem cells, including embryonic, induced pluripotent, tumour and normal adult stem cells [126]. Organoids produce a relevant and highly adaptable model cancer system [127]. They are grown within a 3D matrix system, such as hydrogels, basement membrane extract and Matrigel which have been supplemented with growth factors allowing them to mimic the pancreatic microenvironment [128]. A specialised media is required, with multiple growth factors which mirror the niche organ microenvironments. As organoids are derived from stem cells, they display cell heterogeneity after several passages [127]. Like cell lines, organoids can grow indefinitely and can be cryopreserved, so are a useful PDAC model. Growing organoids in vitro also allows for the observation of disease progression, which requires complex imaging systems in PDX models, or in established cell lines, as these are usually produced from late stage tumours [129,130,131]. Organoids can also be developed from tiny volumes of patient tumour, such as fine needle biopsy, and grown to allow the high throughput screening of drugs and drug combinations [132]. The Tuveson group developed a method which allows for the development of organoids from fine-needle biopsies guided by endoscopic ultrasounds [132]. Organoids can also be developed using tissue biobanks. Walsh et al. [133] investigated the morphology, viability and drug response of frozen organoids with both flash freezing, and DMSO frozen organoids. Expression Ki67 and cleaved caspase3 were assessed to determine viability. Both samples that were flash-frozen and frozen slowly with DMSO were viable, indicating that biobanks of tumour samples could potentially be used for the establishment of organoid cultures.

Organoids can also be orthotopically transplanted into mice, this results in the organoid progressing through all stages of tumour development from PanIN to a PDAC tumour, which represents the tumour of origin [134]. In comparison, when a monolayer of cells is transplanted, the cells rapidly become an aggressive carcinoma [113].

### 4.1. Development of PDAC Organoids

Studies outlining the methods for the production of human and mouse pancreatic organoids have been published by multiple groups [135,136,137]. Both Grapin-Botton and Clevers groups developed methods for the production of murine pancreatic organoids in Matrigel. Grapin-Botton [135] developed pancreatic organoids for use as a model for diabetes. This group used dissociated mouse embryonic pancreatic progenitor cells for the development of organoids. These organoids showed both pancreas morphology and differentiation, and the in vitro maintenance of these pancreatic organoids required the activation of both Notch and fibroblast growth factor (FGF) signalling pathways, which recapitulated the in vivo niche signalling pathway within the pancreas. Clevers [136] developed a method which allowed for the propagation of adult murine pancreatic duct cells as organoids. These organoids were embedded in Matrigel, and a cocktail of growth factors, including Rspondin1 and Wnt3a which stimulate the Wnt signalling pathway.

Clevers, in collaboration with the Tuveson lab, developed a method for the establishment, and growth of normal and cancerous pancreatic organoids from human and mouse tissues [134]. This study showed that both tissues could be established using the same conditions, however, human organoids required additional growth factors, such as Wnt3a. Orthotopic implantation of the tumour and normal organoids resulted in full tumour and ductal development. The methods have been used to further PDAC research, by researching the tumour microenvironment, personalised treatment, genetics and testing of novel therapeutics for PDAC.

To prevent differences in drug response due to batch-to-batch variation of media and extracellular matrix (ECM), Georgakopoulos et al. [138] developed a chemically defined, serum free media, and a chemically defined hydrogel, which allows for the development and propagation of human pancreas tissue. Their study found that the organoids retained their ductal morphology, biomarker expression, and genomic integrity after growth for several months.

In previous work in our lab, we have developed and validated a novel method which allows for the simultaneous development of organoids and primary cell lines from a PDX tumour sample. We developed a method for the establishment of organoids from primary cell lines, termed cell line organoids (CLOs). The usefulness of CLOs as PDAC organoid models is highlighted, as they maintain the same stem cell expression, morphological properties, and RNA-sequencing transcriptomic signatures as their matched patient-derived organoids and PDXs. These models provide a manageable, expandable in vitro resource for downstream applications such as high throughput screening, functional genomics, and tumour microenvironment studies [139].

### 4.2. Organoids as PDAC Tumour-Drug Response Predictors

Using organoids as predictors of drug response can facilitate advanced pre-clinical drug discovery and the personalised treatment of PDAC. Hou et al. [140] used four patient-derived organoid lines including two PDAC derived organoids and two cancer associated fibroblast (CAF) organoids for high throughput screening (HTS) of the National Cancer Institute (NCI) approved oncology set, and 3300 clinically approved drugs.

Huang et al. [141] identified a genotype-phenotype relationship where *TP53*^R175H^ induces cytosolic *SOX9* localisation, whereas in normal pancreas *SOX9* is localised in the nucleus. This finding was verified in two independent PDAC cohorts, and cytoplasmic *SOX9* was associated with a higher tumour grade, poor-disease free survival, and poor overall survival. In this study, organoids also showed a poor response to gemcitabine, the mainstay for PDAC treatment with only 30% growth inhibition.

Studies by Romero-Calvo et al. [142] compared the structural and genetic features of organoids with the primary PDAC tumours, and found the organoids had similar morphologic features with the same glandular architecture. The organoids and primary tumours also had the same protein expression, molecular, genomic and transcriptomic profiles. Response to FOLFIRINOX treatment in organoids recapitulated the matched PDX models. Frappart et al. [143] also found that organoids derived from PDX tumours faithfully recapitulated the PDX morphology and protein expression, and predicted PDX drug response.

A number of proof-of-concept clinical trials using PDAC organoids are underway using fine needle biopsies for the development of organoids (ClinicalTrials.gov: NCT03896958, NCT03544255, NCT03990675 and NCT03140592). Tiriac et al. [144] created a patient derived organoid library from primary PDAC tumours, and metastases, with 75% success. This organoid library was screened using gemcitabine, paclitaxel, SN38, 5-FU and oxaliplatin at clinically relevant concentrations, and the organoid library showed a heterogeneous response to chemotherapies. The outcomes of these assays paralleled the patient outcomes in the clinic. On publishing, Tiriac et al. had also performed whole exome sequencing and RNA-seq and developed gene expression signatures to determine improved response to therapies. In addition to establishing organoid cultures from patient biopsies in these clinical trials, the organoids will be used for drug screening as an indicator of response to therapies. These clinical trials are setting the foundation for the use of organoids in the personalised treatment of pancreatic cancer.

### 4.3. Organoids as Models of Tumour Microenvironment

The tumour microenvironment is known to play an important role in PDAC. By nature, PDAC tumours are dense, fibrotic and hypoxic, and combined with the suppression of tumour infiltrating lymphocytes (TILs) by cytokines such as TGFβ and interleukin-10 (IL-10), PDAC is an non-immunogenic tumour [145].

To identify the role of the tumour microenvironment in PDAC, Tsai et al. [146] produced a new, patient matched organoid model containing, primary PDAC organoids, stromal and immune components. The co-culture of the organoids with cancer-associated fibroblasts resulted in an increased IC50 of 3.8 μM compared to 1.8 μM for organoids alone in response to treatment with gemcitabine. This group also described a method for the introduction of lymphocytes into the organoid culture by adding 500,000 CD3+ T lymphocytes per well suspended in 500 μL organoid growth medium. They demonstrated that lymphocytes only infiltrated into the Matrigel containing organoids. The incorporation of lymphocytes into an organoid co-culture would allow for the use of these models in the study of immunotherapies. The development of methods to study the immune system in PDAC may help overcome the disappointing attempts to use immunotherapy in the treatment of this devastating disease. The use of immunotherapy has resulted in increased survival rates in solid tumour cancers such as melanoma, non-small cell lung cancer, and gastric cancers [147]. However, a successful immunotherapy for PDAC has yet to be developed.

A study by Öhlund et al. [148] used organoids to identify the role of pancreatic stellate cells and cancer associated fibroblasts (CAFs) in PDAC tumour microenvironment and tumour progression. CAFs are derived from activated stellate cells and produce desmoplastic stroma, resulting in differences in disease progression and response to therapies. Co-cultures of organoids and CAFs were established, which resulted in activation of the CAFs to make desmoplastic stroma. These findings were also validated in human and mouse tissues.

### 4.4. Organoids for Biomarker Discovery

As well as using PDAC organoids for modelling in vivo drug response, they have also been used for discovering clinically-actionable biomarkers. Huang et al. [149] demonstrated that organoid models recapitulate the glycomics and drug responses observed in PDX models. The basis of this study was for the identification of N-glycans enriched in the glycome of PDAC using organoids. They identified a set of 57 N-glycans represent 50–94% of the relative abundance of all N-glycans detected. They have also developed a method to use organoids as a discovery platform for blood-based biomarkers in PDAC patients, which can be used for the identification of secreted extracellular vesicles in the blood of patients. This method used 4.0 mL of organoid media supernatant and subjected to LC-MS/MS and identified 241 proteins that were at least two-fold higher in tumour organoid extracellular vesicles compared to exocrine organoids and expressed in at least 4 out of the 6 tumour organoid lines.

### 4.5. Modelling Human Diseases with CRISPR-Cas9-Modified Organoids

The discovery of clustered regularly interspaced short palindromic repeats (CRISPR) and CRISPR-associated (Cas) proteins have revolutionised gene editing. These techniques are readily used in germline gene editing for research in vitro in cell lines, and in vivo in zebrafish, mice, pigs and primates, and recently have been used in organoid technology [150,151,152,153]. For a more detailed review of CRISPR/Cas9 genome editing in organoids, refer to the review by Driehuis and Clevers [154].

Lee et al. [155] edited *KRAS*^G12V^ and *ERBB2*, and inactivated *TP53*, *CDKN2A*, and *SMAD4* by lentiviral delivery of CRISPR-Cas9 into pancreatic ductal organoids. Non-transformed or *KRAS*-mutated ductal organoids ceased proliferation after several passages. *KRAS*, *TP53*, *CDKN2A* and *SMAD4*; and *KRAS*, *TP53*, *CDKN2A*, *SMAD4*, and *ERBB2* CRISPR edited ductal organoids propagated exponentially at least for 4 months. Upon orthotopic xenotransplantation to immunodeficient mice, these ductal organoids developed lesions resembling PanINs, but not PDAC. Seino et al. [156] used a similar CRISPR-Cas9 based method to show the stepwise tumorigenesis of PDAC, and a loss of niche stem cell factor dependence during tumour progression.

### 4.6. Advantages/Limitations of Organoids

The future of organoids in the treatment of PDAC includes their use in personalised medicine including next generation sequencing of the tumour and using organoids for the screening of therapeutics for the identification of the best therapy for patients [157]. Organoids produce an unlimited supply of material for study, therefore reducing the need for animal studies, helping with the implementation of Article 4 of EU Directive 2010/63/EU, which describes the requirements of the 3Rs (Replacement, Reduction and Refinement) that aims to improve the welfare of animals in research [158]. Organoids are derived from stem cells and can form many different cell types and contain a much more realistic mixture of cells for in vitro testing. However, there are several limitations to organoids, including the difficulty in obtaining patient samples. Culturing organoids is laborious, with the need for specialist training and expensive. The use of a 3D matrix environment requires specialist approaches for sample handling, manipulation and functional assays. Finally, in order to analyse in organoid structures, novel imaging and quantitative analysis techniques must be implemented. A comparison of the advantages and disadvantages of organoids, and other PDAC models are outlined in Table 3.

## 5. Conclusions

Advanced pancreatic cancer research has allowed for the identification of hereditary disease variants associated with PDAC, and the discovery of driver and passenger somatic mutations which occur during the progression of the disease. However, little progress has improved patient outcomes and overall survival. Validation of genomic variants identified through GWAS and pathway analysis studies will allow for the identification of those at risk of developing PDAC. However, laboratory models traditionally used to research PDAC, including established and primary cell lines, PDX and GEMM are not representative of how PDAC grows in the patient. Organoids have emerged as a physiologically relevant in vitro model to study cancer. Organoids can be used for both translational research, and for the development of personalised treatment of patients. Additionally, CLOs a novel organoid model, provide a manageable, expandable resource allowing for the use of an organoid model for PDAC research. The future of PDAC research, and the increased survival of patients will be the result of the validation of genomic variants, their influence on disease development, progression and response to therapy in disease appropriate models such as organoids.

## Figures and Tables

**Table 1:** Familial Cancer Syndromes associated with an increased risk of developing pancreatic ductal adenocarcinoma (PDAC). The table includes increased risk, genes associated with syndrome/disease, pathways associated with syndrome/disease and pathway function.

	PJS ^1^	Pancreatitis	FAMMM ^2^	Lynch Syndrome	HBOC ^3^	FAP ^4^
**Increased Risk**	132-fold	69-fold	13–22-fold	8.6-fold	3.5–10-fold	4.5–6-fold
**Genes**	*STK11/LKB11*	*PRSS1* *SPINK1* *CFTR*	*CDNK2A*	*MLH1* *MSH2* *MSH6* *PMS2*	*BRCA1* *BRCA2* *PALB2*	*APC*
**Pathways**	AMPK/mTOR	Trypsin	Retinoblastoma	Mismatch repair	Homologous recombination repair	*Wnt* signalling
**Pathway Function**	Cell growth Polarity Metabolism	Auto-activation of trypsin	G1 to S-phase checkpoint	Maintenance of genomic stability	Repair of double-strand breaks in DNA	Regulation of gene transcription

^1^ Peutz-Jegher Syndrome; ^2^ Familial atypical multiple mole and melanoma syndrome; ^3^ Hereditary Breast and Ovarian Cancer syndrome; ^4^ Familial adenomatous polyposis.

**Table 2:** Gene sets/pathways identified for risk of developing PDAC from pathway analysis studies [62,63].

Pathway/Gene Set	Pathway Reference	Study	Pathway *p*-Value
Maturity onset diabetes of the young	KEGG	[63]	5.10 × 10^−7^
Regulation of Beta cell development	REACTOME	[63]	1.92 × 10^−6^
Breast Cancer 17Q11 Q21 amplicon ^1^	NIKOLSKY	[63]	2.00 × 10^−6^
Role of EGF Receptor Transactivation by GPCRs in Cardiac Hypertrophy	BIOCARTA	[63]	3.79 × 10^−6^
*ATM* Pearson Correlation Coefficient (PCC) Network ^2^	PUJANA	[63]	1.25 × 10^−5^
Pancreas development		[62]	2.0 × 10^−6^
*Heliobacter pylori* lacto/neolacto		[62]	1.6 × 10^−5^
Hedgehog		[62]	0.0019
Th1/Th2 immune response		[62]	0.019
Apoptosis		[62]	0.023

^1.^ Genes within amplicon 17q11-q21 identified in a copy number alterations study of 191 breast tumour samples. ^2.^ Gene network transcripts whose expression positively correlated with *ATM* gene in normal tissues.

**Table 3:** An overview of the advantages and disadvantages of currently available PDAC models.

Model	Representativeness of Patient Sample?	Usage	Maintenance	Success Rates Growth Rate	Cost
Established Cell Lines	Homogenic [88] Undergo genetic modifications [86] Fail to recapitulate the physiology of tumours	High throughput testing	Low maintenance Fast growing	Fast growing Commercially available	Low cost
Primary Cell Cultures	Heterogenous [96] Early passage number [96] Representative of original tumour [96]	High throughput testing	Low maintenance Only grow for a limited number of passages [96]	Some commercially available lines Difficult to establish [97]	Low cost
Organ-on-chip	Heterogenous [101] Allows for the study of the interactions of multiple cell/organ types [98]	Low throughput testing	Medium maintenance	High success rates	Chips are expensive High usage of media and drugs
Organoids	Heterogenous [127] Tumours retain heterogeneity, genetics, and histological characteristics [142]	High throughput testing	Medium maintenance	Medium growing High success rates Established from small volumes of tumour [132]	Expensive ECM and media
PDX ^1^	Tumours retain heterogeneity, genetics, and histological characteristics [103] Replacement of human stroma with murine stroma [108] Orthotopic tumours in correct microenvironment [104]	In vivo and ex vivo drug testing	High maintenance Requires specialist training, and multiple licenses [106]	Slow growing (up to four months) Medium success rates	Expensive to maintain
GEMM ^2^	Tumours in correct microenvironment [109] Immune cells present [112]	In vivo and ex vivo drug testing Testing of immune targeted therapies	High maintenance Requires specialist training, and multiple licences	High success rates Slow growing—(up to 16 weeks)	Commercially available

^1^ Patient Derived Xenografts; ^2^ Genetically Engineered Mouse Models
